# MicroRNA regulation of CYP 1A2, CYP3A4 and CYP2E1 expression in acetaminophen toxicity

**DOI:** 10.1038/s41598-017-11811-y

**Published:** 2017-09-26

**Authors:** Pritmohinder Gill, Sudeepa Bhattacharyya, Sandra McCullough, Lynda Letzig, Prasun J. Mishra, Chunqiao Luo, Harsh Dweep, Laura James

**Affiliations:** 1 Department of Pediatrics, University of Arkansas for Medical Sciences, Little Rock, AR 72202 USA; 2 Arkansas Children’s Research Institute, Little Rock, AR 72202 USA; 3 Department of Biochemical and Cellular Pharmacology, Genentech, 1, DNA Way, South San Francisco, California 94080 USA; 4 The Wistar Institute, 3601 Spruce St, Philadelphia, Pennsylvania 19104 USA

## Abstract

MicroRNAs (miRNAs) that regulate the cytochrome P-450 isoforms involved in acetaminophen (APAP) toxicity were examined in HepaRG cells treated with APAP (20 mM). *In-vitro* studies found that APAP protein adducts were increased at 1 h, followed by ALT increases at 12 and 24 h. CYP1A2, CYP3A4 and CYP2E1 mRNA levels were decreased, while miRNAs were increased for miR-122-5p, miR-378a-5p, miR-27b-3p at 6 h and miR-125b-5p at 12 h and miR-27b-3p at 24 h. Putative miRNA binding sites on the 3′UTRs of the CYPs were identified *in-silico*. Overexpression of miR-122-5p and miR-378a-5p in cells suppressed protein expression of CYP1A2, CYP3A4 and CYP2E1. Luciferase reporter assays confirmed the interaction between miR-122 and the 3′UTR of the CYP1A2 and CYP3A4. Thus, the *in-vitro* experiments showed that miR-122-5p and miR-378a-5p upregulation were associated with translational repression of CYPs. Serum samples of children with APAP overdose had significant elevation of miR-122-5p, miR-378a-5p, miR-125b-5p and miR-27b-3p, compared to healthy controls and receiver operator curves of the miRNAs had AUCs of 91 to 100%. Collectively, the data suggest that miRNA elevations in APAP toxicity represent a regulatory response to modify CYP1A2, CYP3A4 and CYP2E1 translation due to cellular stress and injury.

## Introduction

MicroRNAs (miRNAs, miRs) are 18-25-nucleotides short non-coding RNAs which regulate gene expression^1–3^ through mRNA degradation or the inhibition of protein translation^4,5^. Previous reports have described a role for microRNAs (miRNAs) in the regulation of drug metabolizing enzymes (DMEs)^6–8^. Characterizing miRNA profiles in relationship to cytochrome P450 (CYP) expression for DMEs relevant to APAP toxicity is important to further understanding mechanisms of toxicity and may have relevance for understanding individual variability in susceptibility to drug toxicity, as metabolism is an early, initiating event in the development of APAP hepatotoxicity. A number of laboratories using murine models of APAP toxicity have reported the upregulation of liver enriched miR-122 in association with hepatotoxicity^9–12^ and several researchers have reported elevation of miR-122 in clinical samples of subjects with acetaminophen toxicity^13–18^. Despite this literature, the relationship of miRNAs to DMEs relevant to APAP toxicity (i.e., CYP1A2, CYP3A4 and CYP2E1)^19–23^ is not well understood. In the present study, the relationship of CYP-regulating miRNAs to CYP1A2, CYP3A4 and CYP2E1 gene expression in APAP exposed HepaRG cells was evaluated and compared to indicators of APAP toxicity and metabolism^24,25^. HepaRG cells have high expression and activity levels for DMEs^26–28^ and have been previously used in studies of APAP toxicity^29–31^. In addition, levels of miRNAs were examined in clinical samples obtained from APAP overdose subjects and compared to common clinical indicators of liver injury and APAP toxicity.

## Results

### Time course and dose response; ALT, APAP protein adducts, and CYP-binding miRNAs in HepaRG cells

**Figure 1:**
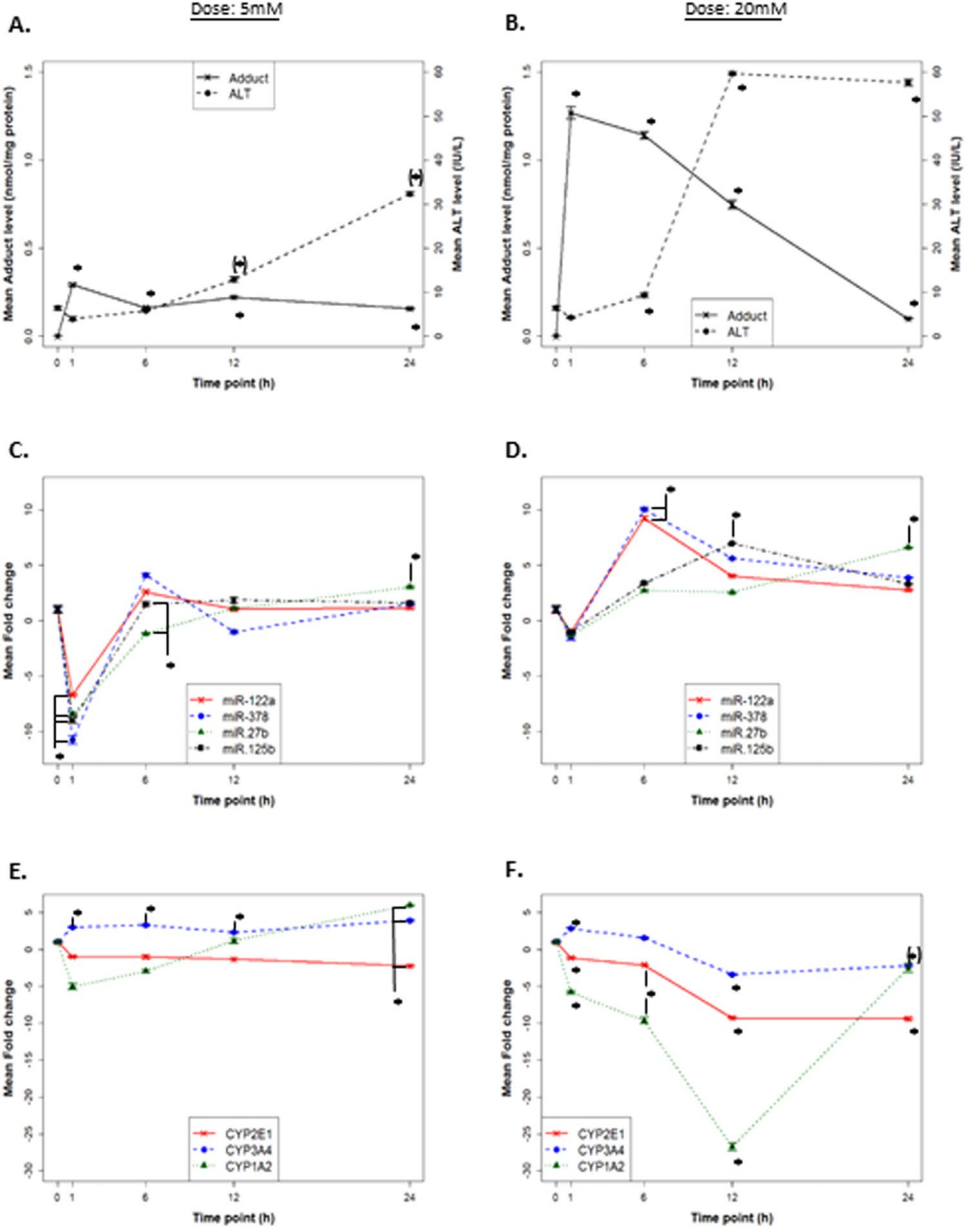
Time and dose-dependent changes in HepaRG cells with APAP treatment for ALT, APAP protein adducts, miRNA (miR-122-5p, miR-378a-5p, miR-27b-3p, and miR-125b-5p and mRNA levels (CYP2E1, CYP3A4, and CYP1A2) levels. Effect of 5 and 20 mM APAP treatment of HepaRG cells at 1, 6, 12 and 24 h time points. (**A** and **B**) Show levels of ALT and APAP protein adducts in cell medium; *p < 0.05 compared to controls for APAP 5 mM and 10 mM. Parenthesis around the asterisk denotes significance of ALT for 5 mM APAP treatment at 12 and 24 h. (**C** and **D**) Depict miRNA expression in cell medium. Data were normalized for HepaRG cell culture medium with Let-7d and spiked-in C. elegans miR-39; *p < 0.05 compared to controls. (**E** and **F**) Describe CYP1E2, CYP1A2 and CYP3A4 mRNA levels determined by qRT-PCR. TaqMan assays were duplexed with GAPDH to normalize the mRNA expression. Parenthesis around the asterisk differentiate 24 h CYP3A4 from CYP1A2; *p < 0.05 compared to controls. “h” Denotes hour. Error bars represent Standard error of the mean.

Dose and response studies were conducted in cells to evaluate the temporal relationships among toxicity (ALT elevation), APAP protein adducts (an indicator of oxidative metabolism) and miRNA profiles. As shown in Fig. 1A, APAP protein adducts were increased at 12 and 24 h in media from cells exposed to APAP 5 mM (12.9 IU/L + 0.57 and 32.3 IU/L + 0.4, respectively; *p < 0.050), compared to control cells (6.4 IU/L + 0.44). Consistent with previous data^29^, a dose response pattern was observed for APAP protein adducts in the APAP 5 and 20 mM exposed cells (Fig. 1B). Figure 1A,B demonstrates that ALT levels increased over time in both dose groups at 24 h (*p < 0.05).

Cells treated with 5 or 20 mM APAP released miR-122-5p, miR-378a-5p, miR-27b-3p, and miR-125b-5p into media as a function of both APAP dose and time. Consistent with the ALT and adduct profiles described above, the most marked changes in miRNA profiles were observed in the APAP 20 mM cells. Figure 1C shows down-regulation of all miRNAs at 1 h in the APAP 5 mM cells, followed by elevation of miR-122-5p at 6 h, miR-378a-5p at 6 h, and miR-27b-3p at 24 h. In contrast, the APAP 20 mM cells (Fig. 1D) had a significant elevation of miR-122-5p at 6 h, miR-378a-5p at 6 h, miR-125b-5p at 12 h, and miR-27b-3p at 24 h. Thus, the data demonstrate that HepaRG cells generate dose-response and temporal data for toxicity and oxidative drug metabolism endpoints known to be important in models of APAP toxicity. Furthermore, miRNA profiles were distinct between the 5 (“low dose”) and 20 mM (“high dose”) exposed cells.

### Effect of APAP treatment on CYP2E1, CYP1A2 and CYP3A4 mRNA expression

To understand temporal relationships between miRNA profiles and gene expression for DME important in APAP toxicity, gene expression of CYP2E1, CYP1A2 and CYP3A4 was examined in cells exposed to APAP 5 mM or 20 mM. As shown in Fig. 1E, CYP2E1 mRNA expression in cells treated with 5 mM APAP was down regulated by 2.2 fold (p < 0.05) at 24 h. Transient down-regulation of CYP1A2 mRNA expression was observed with 5 mM APAP at 1 h (p < 0.05; Fig. 1E), while increased expression was apparent at 24 h. CYP3A4 mRNA expression was increased at all-time points for the APAP 5 mM (Fig. 1E),while the APAP 20 mM cells had down regulation of CYP2E1 at 6, 12 and 24 h (*p < 0.05, Fig. 1F). In contrast, the 20 mM APAP dose produced marked down regulation of CYP1A2 at all-time points, with a 26 fold decrease by 12 h (*p < 0.05, Fig. 1F). Moreover, cells exposed to 20 mM APAP had decreased CYP3A4 mRNA expression at 12 and 24 h (*p < 0.05).

### miRNA target sites located on promoters, 5′UTR, CDS and 3′UTR of CYP genes

The miRWalk2.0 *in-silico* meta-analysis showed that hsa-miR-122-5p, hsa-miR-378a-5p, hsa-miR-27b-3p and hsa-miR-125b-5p have target sites within the 3′UTR region of CYP1A1, CYP1A2, CYP3A4 and CYP2E1 genes. Moreover, a total of 47 interactions were identified between the CYP1A1, CYP1A2, CYP3A4 and CYP2E1 genes and the 4 miRNAs (Table S1). Among these interactions, 15, 5, 16 and 11 were detected on the promoter, 5′ UTR, CDS and 3′ UTR regions, respectively. The mRNA-miRNA interactions suggest preferential control at transcriptional, as well as translational levels. The mRNA 5′ and 3′UTR regions were predicted to harbor several miRNA binding sites. Collectively, the *in-silico* analysis suggests cooperativity and multiplicity of miRNA binding sites on gene regulation during APAP toxicity.

### miR-122 targets 3′ UTR of CYP1A2 and CYP3A4

**Figure 2:**
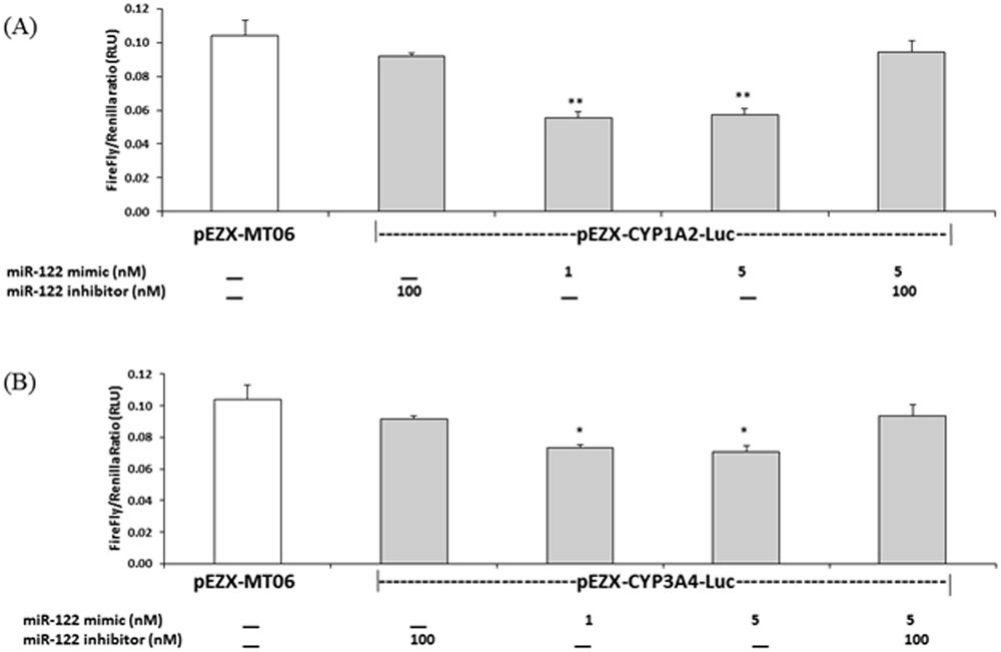
Luciferase assay confirming direct suppression of CYP1A2 and CYP3A4 transcription through 3′UTR binding of miR-122. Effects of miR-122 mimic or inhibitor on luciferase activity in HEK293 cells co-transfected with a firefly luciferase reporter vector containing the CYP1A2 3′UTR-Luc (Fig. 2A) and CYP3A4 3′ UTR-Luc (Fig. 2B). Control plasmid pEZX-MT06 with no 3′UTR, miR-122 mimic (1 nM and 5 nM), miR-122 inhibitor (100 nM). A one-way analysis of variance (ANOVA) with post hoc Dunnett’s test, *p < 0.0196 and **p < 0.006. Values are mean of 2 independent experiments. RLU = relative luciferase units.

To further examine the predicted relationships between miR-122 and CYP1A2 and CYP3A4 (Table S1), HEK293T cells were transfected with 3′UTR luciferase plasmids with miR-122 mimic and inhibitor. Luciferase activity in HEK293T cells expressing the CYP1A2-Luc-3′UTR was inhibited by overexpressing the miR-122 mimic (Fig. 2A) and reporter activity returned to control level (determined by transfection of pEZX-MT06) following transfection with the miR-122 inhibitor. However, overexpression of the miR-122 mimic marginally decreased CYP3A4 3′UTR luciferase activity, while addition of the miRNA-122 inhibitor partially reversed luciferase activity to the level of control plasmid (Fig. 2B). 3′UTR reporter assays demonstrated that the miR-122 mimic reduced luciferase activity, confirming the presence of miR-122-binding sites within the 3′UTR of CYP1A2 and CYP3A4 genes. Mohri and colleagues^32^ previously confirmed the human CYP2E1 3′UTR target for miR-378a.

### Gain-of-function and loss-of function studies: impact of miRNAs on protein expression and toxicity

**Figure 3:**
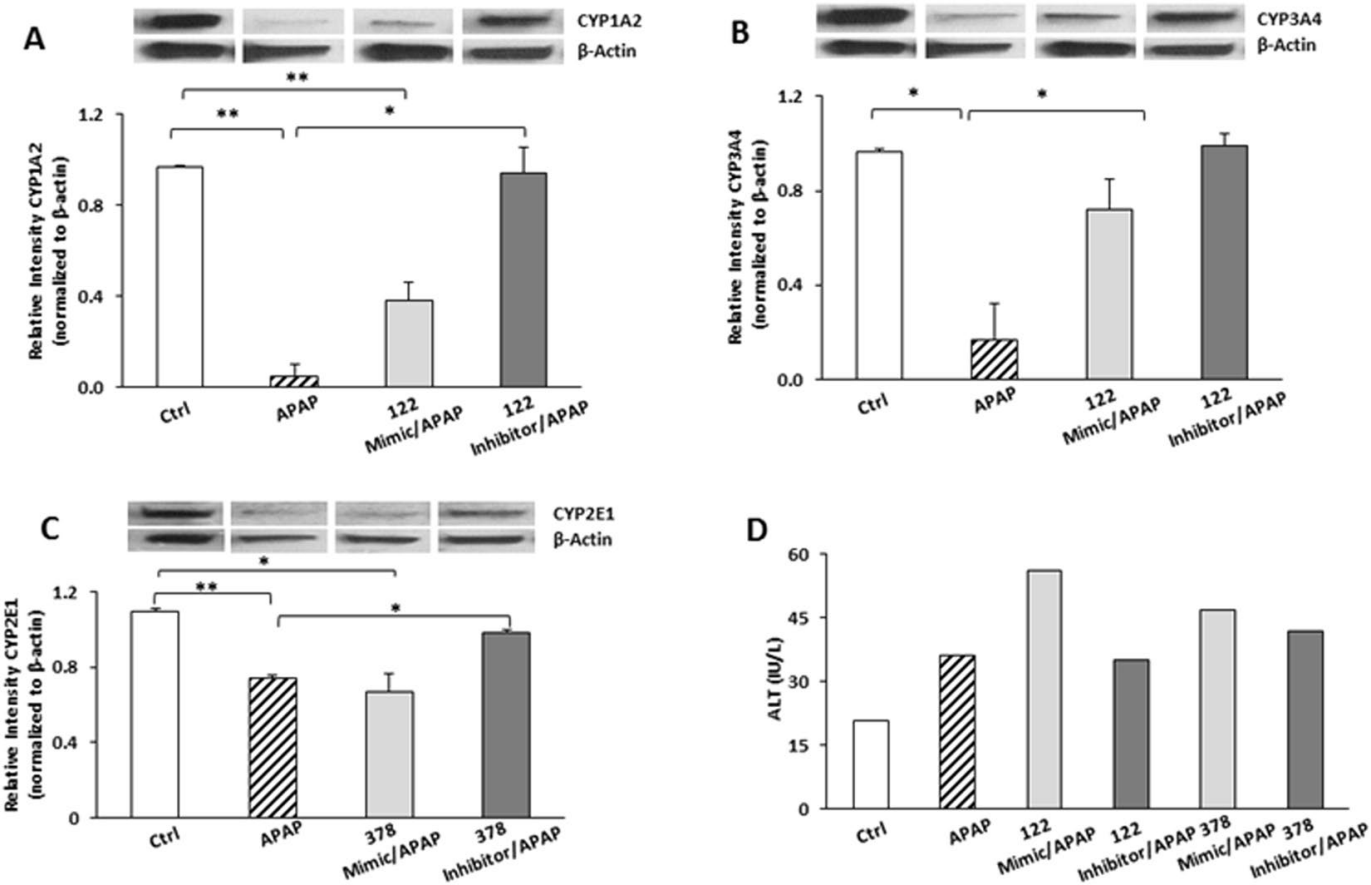
Transfection of miRNA mimic and inhibitor into HepaRG cells and its effect on the protein levels in APAP treated cells (N = 3). Western blot analysis of miR-122 mimic and inhibitor transfected cells treated with APAP for 12 h. (**A**) CYP1A2 and (**B**) CYP3A4: Ctrl = Control untreated; APAP = 20 mM; miR-122 Mimic 5 nM; miR-122 Inhibitor 100 nM. Uncropped image of blots for CYP1A2 and CYP3A4 are shown in Figure S3. Western blot analysis of miR-378 mimic and inhibitor transfected cells (**C**) CYP2E1: Ctrl = Control untreated; APAP = 20 mM; miR-378a Mimic 5 nM; miR-378a Inhibitor 50 nM. Uncropped image of blot for CYP2E1 shown in Figure S4. Data are normalized to β-Actin (*p < 0.05, **p < 0.01) and error bars represent standard error of the mean (SEM). (**D**) ALT levels in miR-122 and miR-378a mimic and inhibitor transfected cells treated with 20 mM APAP for 12 h. “h” denotes hour.

In further studies, the effect of the miR-122-5p and miR-378 mimics and inhibitors on protein expression was examined. CYP1A2 protein expression was reduced by 90% at 12 h in APAP 20 mM cells, compared to controls (Fig. 3A). Transfection with the miR-122-5p mimic attenuated the effect of APAP on CYP1A2 protein expression; CYP1A2 protein expression was reduced by 40% compared to controls. In contrast, transfection of the miR-122-5p inhibitor restored CYP1A2 protein expression to that of controls **(**Fig. 3A).

APAP reduced CYP3A4 protein levels by 83% (Fig. 3B), whereas transfection of cells with the miR-122 mimic reduced CYP3A4 protein levels by only 28%. In addition, transfection of cells with the miR-122 inhibitor restored CYP3A4 expression to that of controls (Fig. 3B). Figure 3C demonstrates that APAP reduced CYP2E1 protein expression by only 26%, whereas cells transfected with the miR-378 mimic had reduced CYP2E1 protein expression by 33%. Transfection with the miR-378 inhibitor restored CYP2E1 protein to that of controls.

Non-APAP exposed cells transfected with mimics and inhibitors of miR-122 and miR-378a had no changes in protein expression for CYP1A2, CYP3A4 and CYP2E1 [Supplementary Figure S1A–F and Figure S2)]. Thus, collectively the data show that gain of function of miR-122 repressed CYP1A2 and CYP3A4, while gain of function of miR-378a repressed CYP2E1 protein levels.

Figure 3D illustrates the effect of mimic and inhibitor transfections on trends in ALT levels. APAP treated cells had increased ALT levels compared to controls. Both the APAP/miR-122 mimic treated cells and the APAP/miR-378 mimic treated cells had higher ALT levels than the APAP only cells. Cells treated with the miR-122 inhibitor or the miR-378 inhibitor had ALT levels comparable to the APAP only cells.

### Elevated levels of APAP protein adducts, ALT, and CYP-binding miRNA in APAP overdose subjects

To examine the clinical relevance of the *in-vitro* data, serum samples from children with APAP overdose were analyzed for APAP protein adducts, ALT, and miRNA. Demographic, toxicity, and APAP protein adduct data are provided in Table 1. Consistent with previous data^25,33,34^, ALT levels and APAP protein adducts were markedly elevated in the serum samples of APAP overdose subjects compared to healthy controls.

**Table 1:** Demographic and clinical parameters of APAP overdose and control subjects (*p < 0.001).

	Control	APAP
N	15	12
Age (years)	7.4 (1.2–16.2)	15.8 (13.6–17.3)
Gender (% Male)	46.7	25
ALT (IU/L)	16.0 (10.0–30.0)	1266.5* (41.0–6072)
APAP Protein Adduct (nmol/mL)	0.0053 (0–0.0097)	1.15* (0.280–3.753)

**Figure 4:**
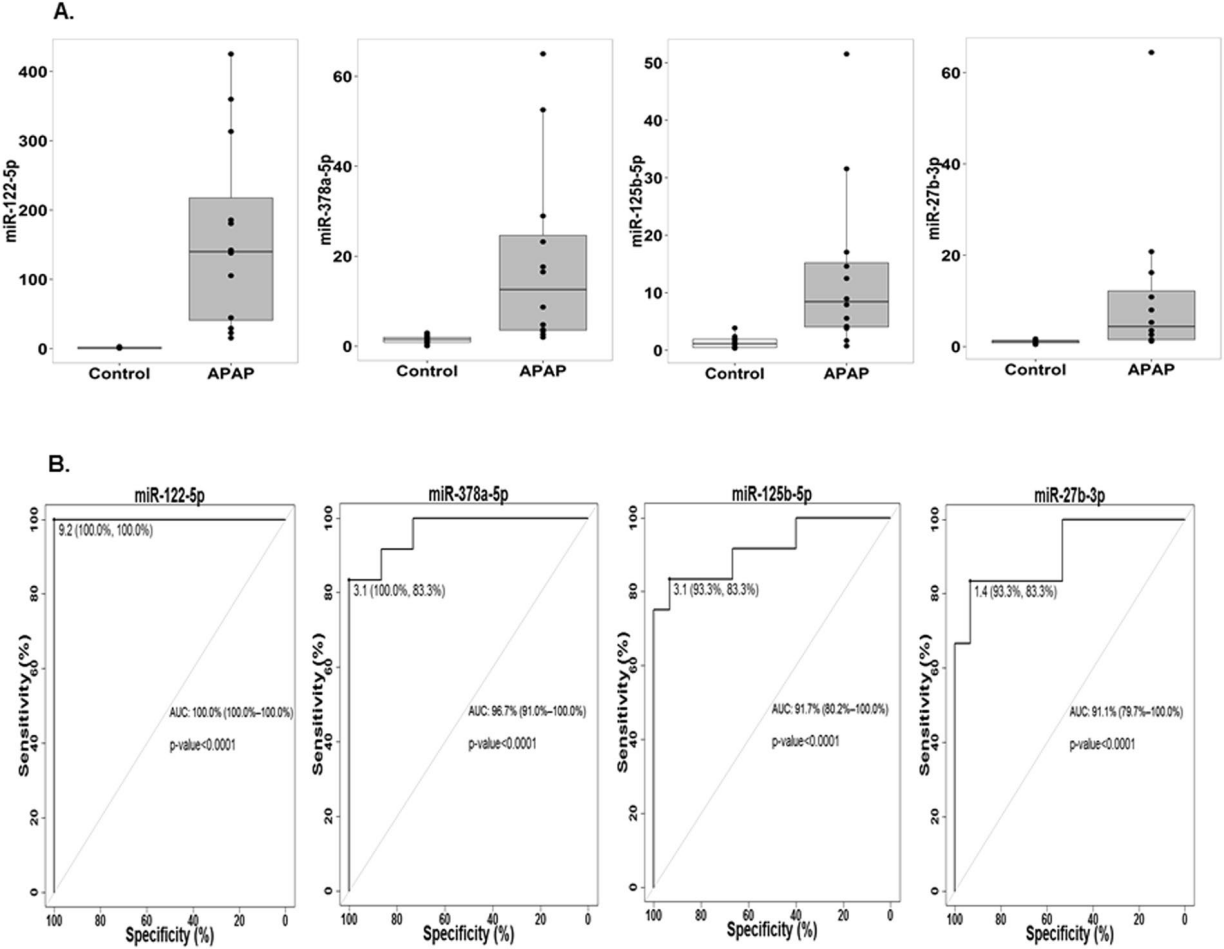
Box plots and ROC curves for miR-122-5p, miR-378a-5p, miR-125b-5p and miR-27b-3p. (**A**) Depicts box plots of circulating miRNAs in serum from healthy and APAP overdose samples. Data was normalized for miRNA expression with let-7d and spiked C. elegans miR-39. Data were presented as a box plot of the median and range of log transformed relative expression level. The top and bottom of the box represent the 75^th^ and 25^th^ percentile. (**B**) Shows receiver operating characteristics (ROC) curve analysis, indicating diagnostic value of each miRNA in discriminating APAP toxicity.

Box plots shown in Fig. 4A demonstrate elevations of miR-122-5p, miR-378a-5p, miR-125b-5p and miR-27b-3p in APAP overdose subjects compared to the control group (*p < 0.05). Serum samples from the APAP group showed a 140 fold elevation of miR-122-5p compared to the control group, whereas elevations of miR-378a-5p, miR-125b-5p, and miR-27b-3p were 12.6, 8.4, and 4.4 fold higher than the control group.

Receiver Operating Curve (ROC) analysis demonstrated the performance for the miRNAs to discriminate between the APAP overdose and control groups (Fig. 4B). The ROC analysis had an Area Under the Curve (AUC) of 1 for miR-122-5p, a predicted regulator of CYP2E1, CYP1A2 and CYP3A4, while AUCs for miR-378a-5p, miR-125b-5p, and miR-27b-3p were 96.7% (91.0–100%), 91.7% (80.2–100%), and 91.1% (79.7–100%), respectively.

**Figure 5:**
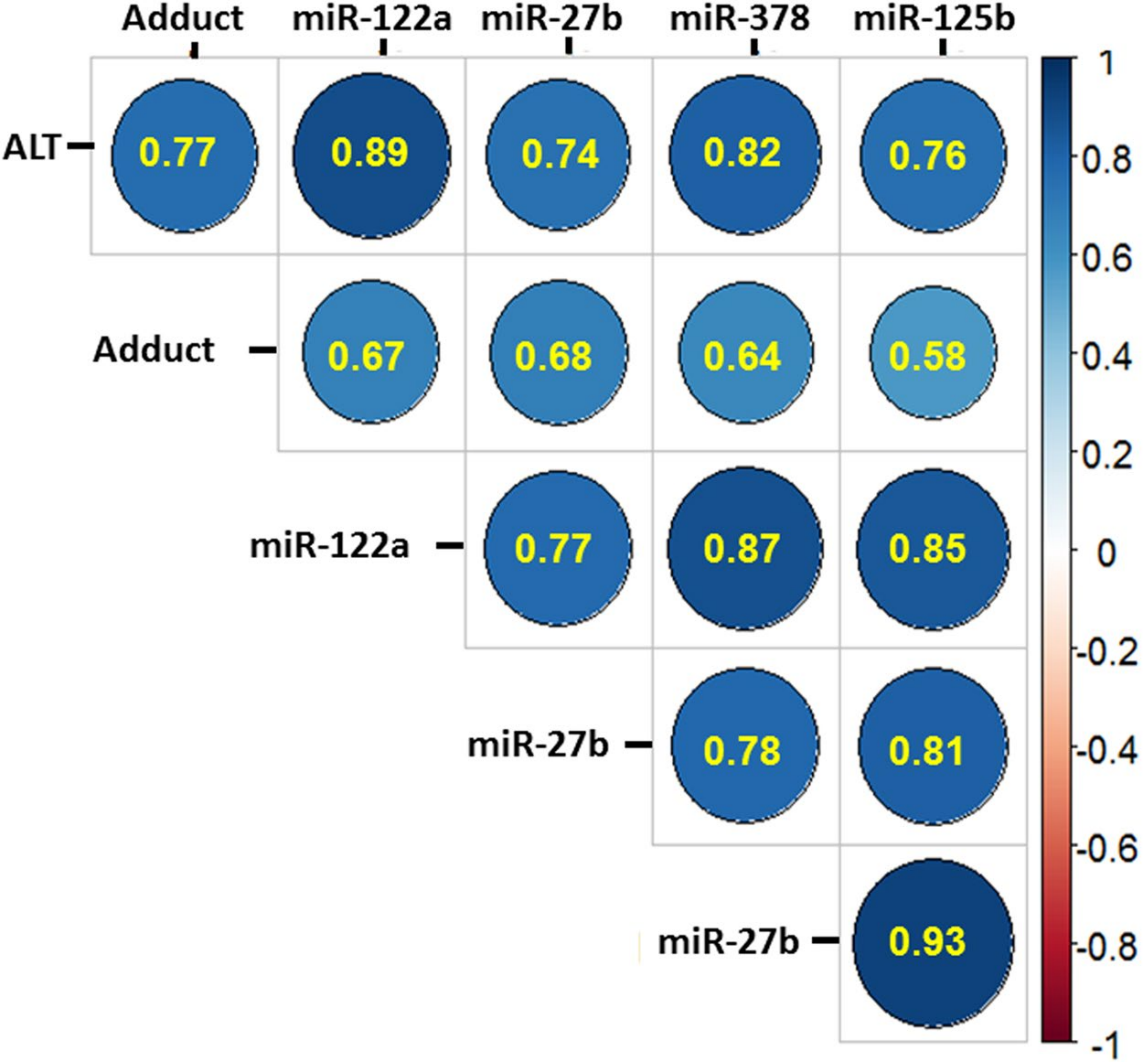
Correlation heatmap showing correlations between ALT, APAP protein adducts and the miRNAs. The correlation coefficients are presented in the circles. On the right is shown a color key for the strength of correlations. Significance level p < 0.05.

Figure 5 provides summary data for correlation coefficients (blue circles) for individual miRNA’s, ALT, APAP protein adducts, and other miRNAs. R values > 0.75 were observed for ALT and miR-122-5p, miR-378a-5p and miR-125b-5p. R values of 0.58-0.68 were observed for APAP protein adducts and miR-122-5p, miR-27b-3p, miR-125b-5p and miR-378a-5p (p < 0.05). Consistent with previous data of Yang and colleagues^17^, a high correlation (R = 0.67) was observed for miR-122-5p and APAP protein adducts^17^.

## Discussion

The present study used HepaRG cells and clinical samples to examine the expression of miRNAs identified to regulate CYPs known to be involved in the metabolism and subsequent development of APAP toxicity^35–37^. Initial *in-silico* analysis determined that miR-122-5p, miR-378a-5p, miR-27b-3p and miR-125b-5p target CYP1A2, CYP3A4 and CYP2E1 genes. Direct suppression of CYP1A2 and CYP3A4 through 3′UTR binding of miR-122 was confirmed by luciferase assays. Gain-of-function and loss-of–function studies demonstrated that miR-122 and miR-378a downregulated CYP 1A2, CYP3A4, and CYP2E1 expression in cells at the translational level. Treatment of cells with two concentrations of APAP (“low” and “high” dose) generated time course toxicity and metabolism data consistent with previous data^29,38^, and indicated differential down regulation of CYP2E1 and CYP1A2 mRNA as a function of APAP dose. In contrast, upregulation of CYP3A4 expression occurred with APAP 5 mM, replicating findings from a recent report^31^, while APAP 20 mM led to down regulation of CYP3A4 expression. APAP treatment resulted in decreased expression of miRNA (miR-122-5p, miR-125b-5p, miR-378a-5p and miR-27b-3p) in cells and increased expression of miRNA’s in media (Fig. 1C,D). These data are consistent with a report by Wang and colleagues^9^ who noted the down-regulation of hepatic miRNAs and subsequent elevation in miRNAs in the plasma of mice treated with a toxic dose of APAP^9^.

Elevations of CYP-regulating miRNAs correlated with elevations of APAP protein adducts in APAP treated cells and in patients with APAP toxicity. Initial studies of miRNA expression in APAP toxicity reported elevations of the liver-specific miR-122-5p and its correlation with ALT elevation^9,10^; miR-122 may also represent a predictive biomarker of APAP liver injury^11^ and resolving liver injury^13,14^. Yamaura *et al*. used a rat model of APAP toxicity and described the elevation of miR-122, as well as miR-192, miR-685, miR-193 and miR-29c^12^. High throughput omic analysis using human APAP overdose samples suggested that miR-122-5p^15–18^, miR-27b-3p^15,16^ and miR-125b-5p^15,17^ were sensitive and noninvasive biomarkers for APAP toxicity. In the present study, we observed a strong association between miR-122-5p, miR-378a-5p, miR-125b-5p, and miR-27b-3p and APAP liver injury (defined by ALT elevation; Fig. 4B).

Prediction algorithms have identified a number of miRNAs that may target CYPs 3′UTR regions^39–41^ in drug metabolism. To our knowledge, the present study is the first to use this approach in APAP toxicity. Of the potential miRNA binding sites identified within the gene regions of the CYPs (CYP2E1, CYP3A4 and CYP1A2) (Table S1) the greatest number of interactions were found for the promoter, CDS and 3′UTR. Taken together in APAP exposed cells down regulation of CYP1A2, CYP3A4 and CYP2E1 (Fig. 1E,F) are mediated by interactions between miRNA binding sites and the CYPs gene regions (promoter, CDS and 3′ UTR). Our findings are in accordance with the previous studies reporting cooperative miRNA-target interactions for 3′UTR and the CDS or 5′UTR region, resulting in strong gene downregulation^42–45^. The analysis also identified region-specific miRNA binding sites (promoter and CDS) for CYP members, consistent with a previous report that suggested that some miRNAs preferentially base-pair to the CDS region, suggesting a key role in rapid inhibition of translation^44^. These interactions, or co-targets, have previously been demonstrated in a number of studies^42–45^, but not in APAP toxicity. As each miRNA can target roughly 200 transcripts^46^, the number of putative interactions may be very large to have an effect on gene regulation in APAP toxicity.

**Figure 6:**
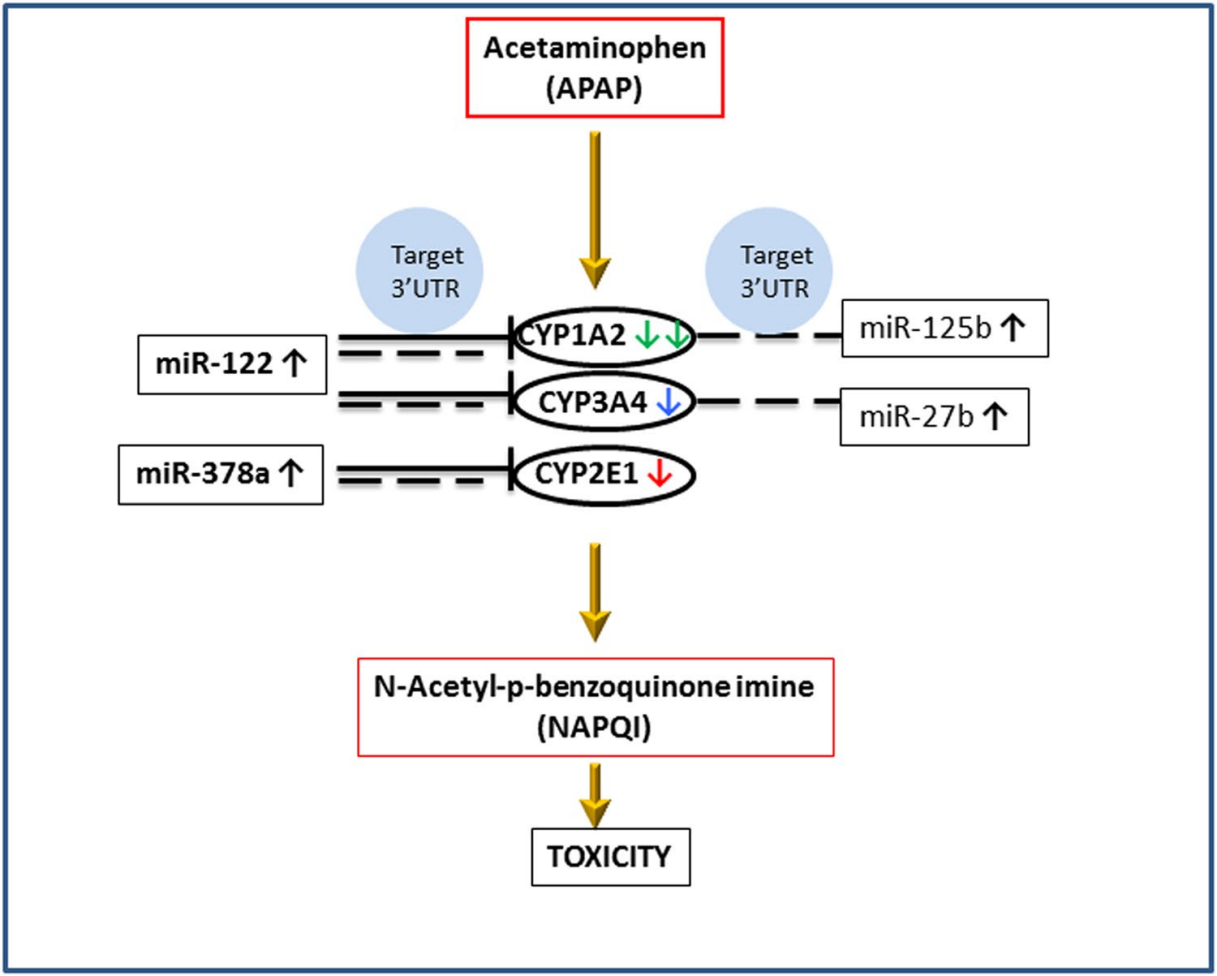
Postulated role of miRNA in APAP toxicity. Schematic illustration of miRNA regulating CYP 450 in APAP toxicity. miRNAs (miR-122, miR-378a, miR-27b and miR-125b) were detected in cell culture medium and serum from APAP over dose subjects. CYP1A2, CYP3A4 and CYP2E1 were detected by qPCR in HepaRG cells and mRNA expression levels match the color coated keys in Fig. 1E,F. Dashed lines show miRNA predicted to target 3′ UTR of CYPs. The bold lines show validation with gain of function experiments for miR-122 and miR-378a mimics in a cellular model.

Target prediction and *in-vitro* functional studies using 3′UTR reporter assays showed that CYP1A2 and CYP3A4 were a direct target of miR-122. The present study also corroborates the work of Mohri and colleagues^32^, who showed that 3′ UTR of CYP2E1 was regulated by miR-378 in HEK293 cells, mainly via translational repression^32^. The data presented herein are consistent with the hypothesis that miR-122-5p and miR-378a-5p upregulation in APAP exposed cells may represent a protective mechanism to lower CYPs expression (Fig. 6) and the formation of the reactive metabolite, N-acetyl-para-quinone imine (NAPQI), and resulting generation of APAP protein adducts. The data also suggest that the predominant effect of miR-122 and miR-378a on CYP1A2, CYP3A4 and CYP2E1 is exerted at the translational level (Fig. 3A–C) and is consistent with recent data from primary cells and tissues from miRNA mutant mice^5^ showing that 48% of target genes are regulated by translational repression. Thus, the data provide new insights into the function of miRNA in the regulation of CYP expression in APAP toxicity and suggests that miRNAs may be a potential target for novel therapeutic approaches in APAP toxicity.

One limitation of the present study its reliance on HepaRG cells which do not fully capture the inherent biologic variability and microenvironment of the human liver. It is generally regarded that APAP toxicity is a direct necrotic event, although signaling cascades invoked by cytokine release from inflammatory cells and other pathways may modulate the toxicity. Since the liver comprises a variety of cell types, further well characterized *in-vivo* studies are necessary to fully understand the miRNA regulated signaling pathways in APAP toxicity.

To summarize, serum miR-122-5p was highly increased in children with APAP overdose, similar to data published in adults. Three other miRNAs (miR-378a-5p, miR-125b-5p, and miR-27b-3p) were also increased, but the relative increase was lower than that of miR-122-5p. Strong correlations were noted for ALT and miR-122-5p, miR-378a-5p and miR-125b-5p (R = 0.76–89, p < 0.05), while moderate correlations were noted for APAP protein adducts and the miRNAs (R = 0.58–0.67, p < 0.05). These elevations may represent cell-to-cell communication in an attempt to minimize toxic events in cells or stimulate hepatocyte repair responses. A better understanding of the function of miRNAs in APAP-induced hepatotoxicity may have relevance for the development of new treatments for APAP toxicity and/or understanding individual susceptibility to drug toxicity. The results provide new insights into the protective role of miR-122 and miR-378a in suppressing the expression of DMEs.

## Methods

### Chemicals and materials

APAP, nuclease free water and ethanol (Molecular grade) were purchased from Sigma-Aldrich (St. Louis, MO). miRNA and RNA isolation, cDNA synthesis and pre-amplification reagents were purchased from Qiagen (Valencia, CA). Collagen coated 24 well plates and phosphate buffered saline were obtained from ThermoFisher Scientific (Carlsbad, CA). The miR-122 mimic, miR-122 inhibitor, miR-378a mimic, miR-378a inhibitor, AllStars Negative control siRNA and Hs Cell Death Control siRNA were purchased from Qiagen (Valencia, CA).

### HepaRG cells

HepaRG cells (ThermoFisher Scientific,Carlsbad, CA) were seeded in base medium per the manufacturer’s protocol. Cell viability was determined as per Trypan blue method and 330,000 cells were seeded per well in collagen-coated 24 well plates. As per protocol, from day 2–9 base medium was supplemented with Tox Medium Supplement (ThermoFisher Scientific, Carlsbad, CA).

### APAP treatment, mRNA/miRNA isolation, cDNA synthesis and quantitative real-time PCR (qRT-PCR)

Cells were incubated with 5 or 20 mM APAP^26,29–31^ on day 7 for 1, 6, 12, or 24 h time points. Isolation of RNA from cells was performed using the miRNeasy Mini Kit (Qiagen, Valencia, CA). Total RNA (500 ng) was used for cDNA synthesis using RT2 First Strand Kit (Qiagen, Valencia, CA). For gene expression analysis, quantitative real-time polymerase chain reactions (qRT-PCRs) were performed using TaqMan Fast Master Mix (ThermoFisher Scientific, Carlsbad, CA) for Cyp1A2 (Hs00167927_m1), Cyp2E1 (Hs00559368_m1) and Cyp3A4 (Hs00604506_m1) and were duplexed with GAPDH (Hs02758991_g1). RNeasy MinElute Cleanup Kit was used for miRNA isolation from cells (Qiagen, Valencia, CA) and cDNA was synthesized with the miScript II RT kit (Qiagen, Valencia, CA). For media samples, the total RNA, including miRNA was isolated from 100 µl media spiked in with C. elegans miR-39 (Ce_miR-39_1) using miRNeasy Serum/Plasma kit (Qiagen, Valencia, CA). cDNA synthesis was performed using the miScript II RT kit (Qiagen, Valencia, CA) with miScript HiSpec buffer. cDNA from cells and media was pre-amplified with miScript PreAMP PCR Kit (Qiagen, Valencia, CA). For miRNA expression analysis, cDNAs from cells and media were used in qRT-PCR reaction for miScript primer assays: hsa-miR-122-5p (MS00003416), hsa-miR-378a-5p (MS00009646), hsa-miR-27b-3p (MS00031668), and hsa-miR-125b-5p (MS00006629), Hs_let_7d_1 (MS00003136), Ce_miR-39_1 (MS00019789) and Hs_SNORD95 (MS00033726) (Qiagen, Valencia, CA). Expression levels of miRNAs and CYP mRNA were measured in triplicate on a QuantStudio 6 Flex Real-Time PCR System (ThermoFisher Scientific, Carlsbad, CA).

### Toxicity assays

Hepatic necrosis was assessed by measurement of alanine aminotransferase (ALT) in media using an Ace Alera Serum Chemistry Analyzer (Alfa Wassermann).

### Human samples

Peripheral blood samples were collected as part of a multicenter investigation of APAP toxicity in children of ages 2–18 years, approved by University of Arkansas for Medical Sciences (UAMS) institutional review board (IRB)^33^. Subjects were categorized as controls (n = 15 healthy children with no use of APAP in the preceding 2 weeks) or APAP overdose (n = 12; children that required hospitalization for treatment of APAP overdose) and informed consent was obtained from all participants per UAMS IRB policy guidelines and all experiments were performed in accordance with the relevant guidelines and regulations. A single blood sample was collected from control subjects, whereas daily samples were collected from the APAP overdose subjects. Blood samples were centrifuged within 30 minutes of collection and the serum was stored at −80 °C. Demographic information available for study subjects included subject age, gender, weight, height, body surface area, past medical history, reason for hospitalization, relevant history concerning recent APAP dosing and concomitant medications. Clinical laboratory results included APAP concentration and ALT determinations.

### Quantitation of APAP protein adducts

APAP protein adducts were analyzed using a method previously published by our laboratory^24,47,48^. Briefly, 100 µl of serum or cell supernatants was gel filtered, hydrolyzed with protease, precipitated, and injected onto an HPLC system. APAP cysteine was resolved on a 150 mm C18 column and detected using an ESA CoulArray electrochemical detector. Concentrations of adducts were determined relative to a standard curve of authentic APAP cysteine and reported as nmoles a-cys/mg protein (*in-vitro* data) and nmol/L a-cys (human serum samples).

### Serum miRNA isolation and cDNA synthesis

From 100 µl serum sample total RNA including miRNA was isolated, reverse transcribed and pre-amplified as mentioned above in section describing isolation from media.

### miRNA expression analysis

The pre-amplified cDNA from cells, media and human sera were used to run qRT-PCR using miScript SYBR Green PCR Kit and miScript Primer Assays for miRNAs (hsa-miR-122-5p, hsa-miR-378a-5p, hsa-miR-27b-3p, and hsa-miR-125b-5p) (Qiagen, Valencia, CA). All samples were run in triplicate on QuantStudio 6 Flex Real-Time PCR System (ThermoFisher Scientific, Carlsbad, CA). For each primer assay, negative controls were run with water and cDNA samples. For no template controls (NTC) a master mix with no cDNA was used. Data normalization was performed where appropriate with exogenous control (Ce-miR-39) and endogenous controls (let-7d, RNU6 and/or SNORD95). Relative quantitation was calculated using the 2^−ΔΔCt^ method.

### microRNA target predictions

miRWalk2.0^49,50^ is a comprehensive database (http://zmf.umm.uni-heidelberg.de/apps/zmf/mirwalk2/generetsys-self.html) that provides *in-silico* meta-analysis of miRNA-target interactions by combining data sets from 13 different prediction algorithms. The complete sequence (promoter, CDS, 5′ and 3′UTR regions) of CYP1A1, CYP1A2, CYP3A4 and CYP2E1 genes was screened to identify binding sites of 4 miRNAs (hsa-miR-122-5p, hsa-miR-125b-5p, hsa-miR-378a-5p, and hsa-miR-27b-3p), previously reported in APAP toxicity studies^3,5,22,36–39,41,42^.

### 3′UTR luciferase reporter assays

To confirm the miRNA-mRNA interactions, human CYP1A2 (HmiT003774) and CYP3A4 (HmiT055335) 3′ UTR target clones in pEZX-MT06 were purchased (GeneCopoeia, Rockville, MD). HEK293T cells were seeded in 96-well plates (3 × 10^4^ cells/well) for 24 h. HEK293T cells were co-transfected in OPTI MEM media (ThermoFisher Scientific, Carlsbad, CA) with 200 ng of 3′ UTR luciferase reporter plasmids and miR-122 mimic, miR-122 inhibitor or negative control plasmid pEZX-MT06 (GeneCopoeia, Rockville, MD) with Attractene transfection reagent (Qiagen, Valencia, CA). Firefly and renilla luciferase activities were measured 24 h after transfection using the Luc-Pair Duo-Luciferase Assay Kit 2.0 (GeneCopoeia, Rockville, MD). Data represent the ratio of firefly luciferase activity to that of Renilla.

### Transfection of microRNA mimics and inhibitors

Mimics and inhibitors of miR-122 and miR-378 were used to overexpress or knockdown miRNA in HepaRG cells. Cells were grown in 24 well plate (see cell culture) and transfected with 5 nM mimic, 50 nM or 100 nM inhibitor using RNAiMAX Transfection Reagent (ThermoFisher Scientific, Carlsbad, CA). At 24 h, the transfection medium was replaced with HepaRG cells Tox Working Medium. At 72 h, cells were cultured in glutathione free media. APAP (20 mM) was added for 12 h and cells were harvested at 96 h post transfection for protein analysis. Transfection efficiency of 65–89% was observed with 5–50 nM concentration of AllStars Hs Cell Death Control siRNA. Preliminary studies found no interference in protein expression with negative control AllStars siRNA.

### Protein isolation and Western blot analysis

After transfections, cells were lysed with ice-cold RIPA lysis buffer (ThermoFisher Scientific, Carlsbad, CA) containing protease inhibitors (Pierce Protease Inhibitor Mini Tablets, Rockford, IL and Mammalian Protease Arrest, GBiosciences, St. Louis, MO) and centrifuged at 12,500 rpm for 10 min at 4 °C. Cell supernatants were used for immunoblots. Proteins were quantified using Pierce BCA Protein Assay Kit (ThermoFisher Scientific, Carlsbad, CA) and 20 ug of protein was loaded on to precast polyacrylamide Bis-tris 10% gels (GenScript, Piscataway, NJ). The membranes were blocked with 5% skim milk and incubated with primary antibodies against CYP2E1, CYP1A2 and CYP3A4 (Abcam, Cambridge, MA) and β-actin (Sigma, St. Louis, MO). Next membranes were incubated with HRP-conjugated secondary antibodies to goat anti-rabbit IgG H&L (Abcam, Cambridge, MA). The protein bands were visualized using the SuperSignal West Pico Chemiluminescent Substrate (ThermoFisher Scientific, Carlsbad, CA) and densitometry was performed with the ImageJ software program (NIH, Bethesda, MD).

### Statistical analysis

Statistical analyses of cellular data used one-way ANOVA with post hoc Dunnett’s test to determine differences between treatment groups and the control. The unpaired Student’s t-test was used for comparison between two cell groups. The non-parametric Mann–Whitney U test was used for pairwise comparisons between clinical groups for toxicity (ALT), metabolism (APAP protein adducts) and miRNA data. Spearman rank correlation analysis was used to assess the pairwise relationships among miRNAs, ALT and APAP protein adducts; data are reported as medians, minimums, and maximums. A p value < 0.05 was considered significant for all analyses. Receiver operating curve (ROC) analysis was used to assess the performance of each miRNA as a binary classifier of toxicity outcomes.

### Disclosure

Dr. James has a patent application pending for the measurement of APAP protein adducts in human blood samples.

## Electronic supplementary material

Supplementary information

## References

[1] Ambros V (2004). The functions of animal microRNAs. Nature.

[2] Bartel DP (2009). MicroRNAs: target recognition and regulatory functions. Cell.

[3] Chen K, Rajewsky N (2007). The evolution of gene regulation by transcription factors and microRNAs. Nat Rev Genet.

[4] Baek D (2008). The impact of microRNAs on protein output. Nature Sep 4;.

[5] Jin H.Y., Xiao C. MicroRNA Mechanisms of Action: What have We Learned from Mice? *Front Genet*. Nov 16; 6, 328. eCollection (2015). 10.3389/fgene.2015.00328 PMC4644800 26635864

[6] Rieger JK (2013). Expression Variability of ADME-Related Micrornas in Human Liver: Influence of Non-Genetic Factors and Association with Gene Expression. Drug Metab Dispos.

[7] Ramamoorthy A (2013). Regulation of microRNA expression by rifampin in human hepatocytes. Drug Metab Dispos.

[8] Yu AM (2016). MicroRNA Pharmacoepigenetics: Posttranscriptional Regulation Mechanisms behind Variable Drug Disposition and Strategy to Develop More Effective Therapy. Drug Metab Dispos Mar.

[9] Wang K (2009). Circulating microRNAs, potential biomarkers for drug-induced liver injury. Proc Natl Acad Sci USA.

[10] Bala S (2012). Circulating microRNAs in exosomes indicate hepatocyte injury and inflammation in alcoholic, drug-induced, and inflammatory liver diseases. Hepatology.

[11] Starckx S (2013). Evaluation of miR-122 and other biomarkers in distinct acute liver injury in rats. Toxicol Pathol.

[12] Yamaura Y (2012). Plasma microRNA profiles in rat models of hepatocellular injury, cholestasis, and steatosis. PLoS One 2012.

[13] Starkey Lewis PJ (2011). Circulating microRNAs as potential markers of human drug-induced liver injury. Hepatology.

[14] Antoine DJ (2013). Mechanistic biomarkers provide early and sensitive detection of APAP-induced acute liver injury at first presentation to hospital. Hepatology.

[15] Ward J (2014). Circulating microRNA profiles in human patients with APAP hepatotoxicity or ischemic hepatitis. Proc Natl Acad Sci USA.

[16] Krauskopf J (2015). Application of High-Throughput Sequencing to Circulating microRNAs Reveals Novel Biomarkers for Drug-induced Liver Injury. Toxicol Sci.

[17] Yang X (2015). Potential of extracellular microRNAs as biomarkers of APAP toxicity in children. Toxicol Appl Pharmacol.

[18] Vliegenthart AD (2015). Comprehensive microRNA profiling in acetaminophen toxicity identifies novel circulating biomarkers for human liver and kidney injury. Sci Rep.

[19] Patten CJ (1993). Cytochrome P450 enzymes involved in APAP activation by rat and human liver microsomes and their kinetics. Chem Res Toxicol.

[20] Snawder JE, Roe AL, Benson RW, Roberts DW (1994). Loss of CYP2E1 and CYP1A2 activity as a function of APAP dose: relation to toxicity. Biochemical and Biophysical Research Communications.

[21] Raucy JL, Lasker JM, Liebler CS, Black M (1989). APAP activation by human liver cytochromes P4502E1 and P4501A2. Arch Biochem Biophys.

[22] Tonge RP (1998). Role of CYP1A2 in the hepatotoxicity of APAP: Investigations using CYP1A2 null mice. Toxicology and Applied Pharmacology.

[23] Laine JE, Auriola S, Pasanen M, Juvonen RO (2009). APAP bioactivation by human cytochrome P450 enzymes and animal microsomes. Xenobiotica.

[24] Muldrew KL (2002). Determination of APAP-protein adducts in mouse liver and serum and human serum after hepatotoxic doses of APAP using high-performance liquid chromatography with electrochemical detection. Drug Metab Dispos.

[25] James LP (2009). Pharmacokinetics of APAP-protein adducts in adults with APAP overdose and acute liver failure. Drug Metab Dispos.

[26] Aninat C (2006). Expression of cytochromes P450, conjugating enzymes and nuclear receptors in human hepatoma HepaRG cells. Drug Metab Dispos.

[27] Hart SN (2010). A comparison of whole genome gene expression profiles of HepaRG cells and HepG2 cells to primary human hepatocytes and human liver tissues. Drug Metab Dispos.

[28] Rogue A (2012). Interindividual variability in gene expression profiles in human hepatocytes and comparison with HepaRG cells. Drug Metab Dispos.

[29] McGill MR (2011). HepaRG cells: a human model to study mechanisms of APAP hepatotoxicity. Hepatology.

[30] Tobwala S, Khayyat A, Fan W, Ercal N (2015). Comparative evaluation of N-acetylcysteine and N-acetylcysteineamide in acetaminophen-induced hepatotoxicity in human hepatoma HepaRG cells. Exp Biol Med.

[31] Rodrigues RM (2016). Toxicogenomics-based prediction of acetaminophen-induced liver injury using human hepatic cell systems. Toxicol Lett.

[32] Mohri T (2010). Human CYP2E1 is regulated by miR-378. Biochem Pharmacol.

[33] Bhattacharyya S (2014). Targeted liquid chromatography-mass spectrometry analysis of serum acylcarnitines in APAP toxicity in children. Biomark Med.

[34] James L (2015). Comparison of Bile Acids and Acetaminophen Protein Adducts in Children and Adolescents with Acetaminophen Toxicity. PLoS One.

[35] Lee SS (1996). Role of CYP2E1 in the hepatotoxicity of APAP. J Biol Chem..

[36] Manyike PT, Kharasch ED, Kalhorn TF, Slattery JT (2000). Contribution of CYP2E1 and CYP3A to APAP reactive metabolite formation. Clin Pharmacol Ther.

[37] Sarich T (1997). The effect of omeprazole pretreatment on APAP metabolism in rapid and slow metabolizers of S-mephenytoin. Clin Pharmacol Ther.

[38] Xie Y (2015). Time course of acetaminophen-protein adducts and acetaminophen metabolites in circulation of overdose patients and in HepaRG cells. Xenobiotica.

[39] Singh D, Kashyap A, Pandey RV, Saini KS (2011). Novel advances in cytochrome P450 research. Drug Discov Today.

[40] Swart M, Dandara C (2014). Genetic variation in the 3′-UTR of CYP1A2, CYP2B6, CYP2D6, CYP3A4, NR1I2, and UGT2B7: potential effects on regulation by microRNA and pharmacogenomics relevance. Front Genet.

[41] Ramamoorthy A, Skaar TC (2011). In silico identification of microRNAs predicted to regulate the drug metabolizing cytochrome P450 genes. Drug Metab Lett.

[42] Fang Z, Rajewsky N (2011). The Impact of miRNA Target Sites in Coding Sequences and in 3′UTRs. PLoS ONE.

[43] Lee I (2009). New class of microRNA targets containing simultaneous 5′-UTR and 3′-UTR interaction sites. Genome Res.

[44] Hausser J, Syed AP, Bilen B, Zavolan M (2013). Analysis of CDS-located miRNA target sites suggests that they can effectively inhibit translation. Genome Res.

[45] Forman JJ, Coller HA (2010). The code within the code: microRNAs target coding regions. Cell Cycle.

[46] Krek A (2005). Combinatorial microRNA target predictions. Nat Genet.

[47] Khandelwal N (2011). Unrecognized APAP toxicity as a cause of indeterminate acute liver failure. Hepatology.

[48] Roberts DW (2017). An Immunoassay to Rapidly Measure Acetaminophen Protein Adducts Accurately Identifies Patients With Acute Liver Injury or Failure. Clin Gastroenterol Hepatol.

[49] Dweep H, Gretz N (2015). miRWalk2.0: a comprehensive atlas of microRNA-target interactions. Nat Methods.

[50] Dweep H, Sticht C, Pandey P, Gretz N (2011). miRWalk–database: prediction of possible miRNA binding sites by “walking” the genes of three genomes. J Biomed Inform.

