# Supplementary material for: MicroRNA regulation of CYP 1A2, CYP3A4 and CYP2E1 expression in acetaminophen toxicity
Source: Sci Rep. 2017 Sep 26;7:12331. doi: 10.1038/s41598-017-11811-y (PMC5614957; doi:10.1038/s41598-017-11811-y)
Supplement: Supplementary file 1 — Supplementary information [file 41598_2017_11811_MOESM1_ESM.docx]

# Supplement To

# MicroRNA regulation of CYP 1A2, CYP3A4 and CYP2E1 expression in acetaminophen toxicity

Pritmohinder Gill^1,2+*^, Sudeepa Bhattacharyya^1, 2+^, Sandra McCullough^1, 2^, Lynda Letzig^1,2^ Prasun J. Mishra^3^, Chunqiao Luo^2^, Harsh Dweep^4^, Laura James^1,2^

^1^Department of Pediatrics, University of Arkansas for Medical Sciences, Little Rock, AR 72202, USA

^2^Arkansas Children’s Research Institute, Little Rock, AR 72202, USA

^+^Authors contributed equally to the work; E-Mails: [PSGill@uams.edu](mailto:PSGill@uams.edu) (PG); [SBhattacharyya2@uams.edu](mailto:SBhattacharyya2@uams.edu) (SB); [McCulloughSandraS@uams.edu](mailto:McCulloughSandraS@uams.edu) (SM); [LetzigLyndaG@uams.edu](mailto:LetzigLyndaG@uams.edu) (LL); [CLuo@uams.edu](mailto:CLuo@uams.edu) (CL); [JamesLauraP@uams.edu](mailto:JamesLauraP@uams.edu) (LJ)

^3^Small Molecule Drug Discovery and Biochemical and Cellular Pharmacology Department

Genentech, 1 DNA Way, Building 11, Room -115, MS 224 South; San Francisco, CA 94080; [mishrapj@gmail.com](mailto:mishrapj@gmail.com) (PJM)

^4^The Wistar Institute, 3601 Spruce St, Philadelphia, Pennsylvania 19104, USA^;^ hdweep@wistar.org (HD)

*Corresponding author:

Dr. Pritmohinder Gill

[PSGill@uams.edu](mailto:PSGill@uams.edu)

Telephone: +1(501)364-1418

FAX: +1(501)364-3654

**Supplementary Figures (Figure S1-4)**

**Supplementary Table 1 (Table S1)**

**Figure S1: Transfection of mimic and inhibitor of miR-122 and miR-378a into HepaRG cells with no APAP.** Panel A-D: CYP1A2 and CYP3A4 protein expression with miR-122 mimic and inhibitor. Panel E-F: CYP2E1 protein expression with miR-378a mimic and inhibitor. Values are mean of 2 independent experiments. Protein expression was normalized to β-actin. Error bars represent Standard error of the mean.

**
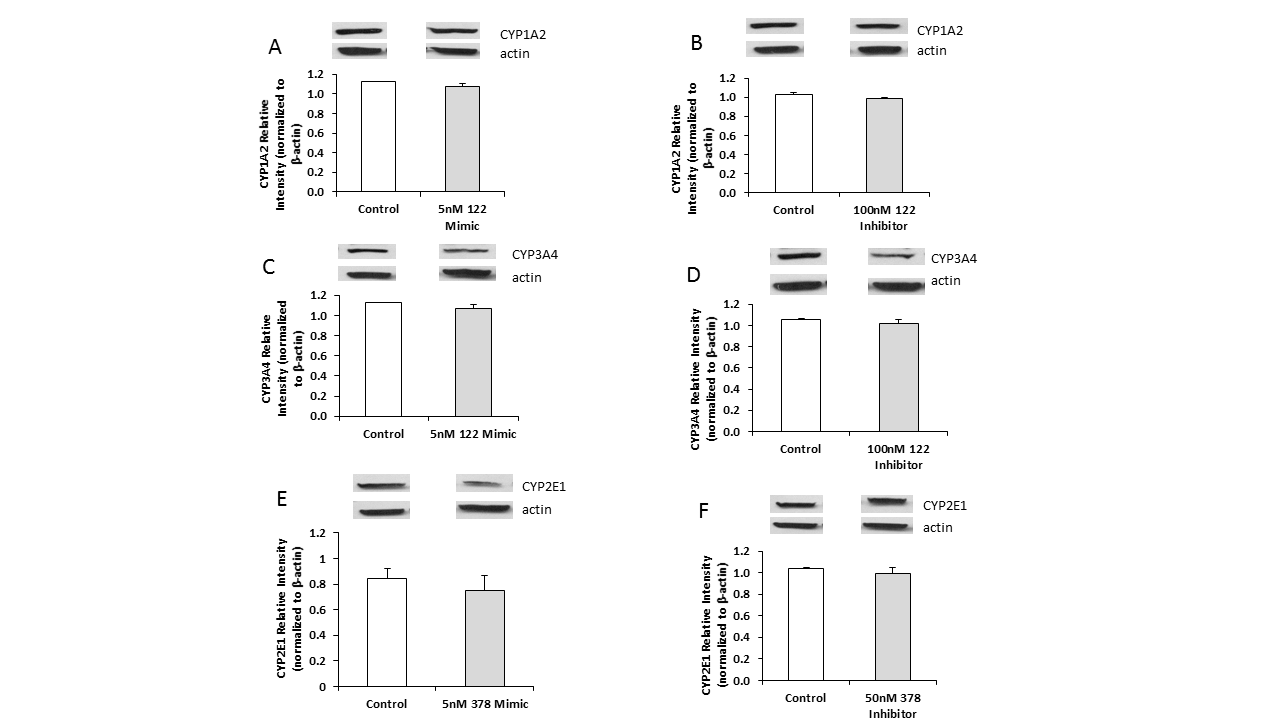
**

**Figure S2: Transfection of mimic and inhibitor of miR-122 and miR-378a into HepaRG cells with no APAP.** Uncropped images of Figure S1 and rectangle drawn on bands were used to create Figure S1. CYP1A2 and CYP3A4 protein expression with miR-122 mimic and inhibitor. CYP2E1 protein expression with miR-378a mimic and inhibitor.

**
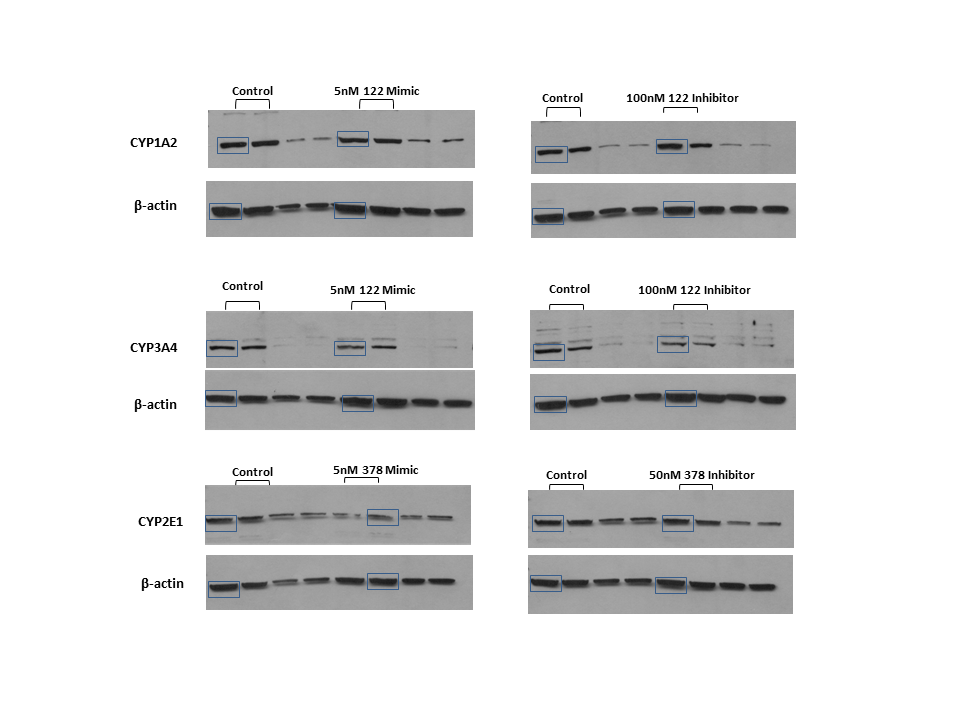
**

**Figure S3: Uncropped scans of Figure 3 (A), and 3 (B). Western blots showing CYP1A2 and CYP3A4 expression in HepaRG cells transfected with miR-122 mimic and inhibitor. APAP= acetaminophen; inhib=inhibitor.**

**
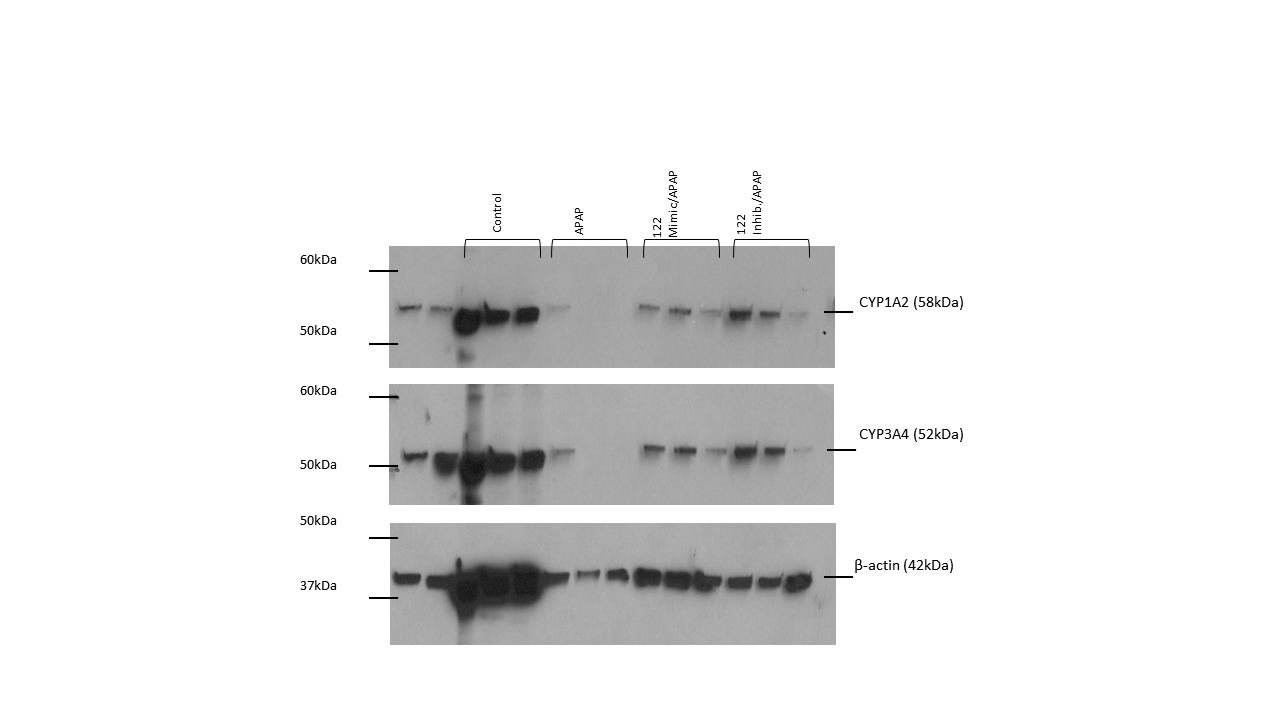
**

**Figure S4: Uncropped scans of Figure 3 (C). Western blots showing CYP2E1 expression in HepaRG cells transfected with miR-378a mimic and inhibitor. APAP= acetaminophen; inhib=inhibitor.**

**
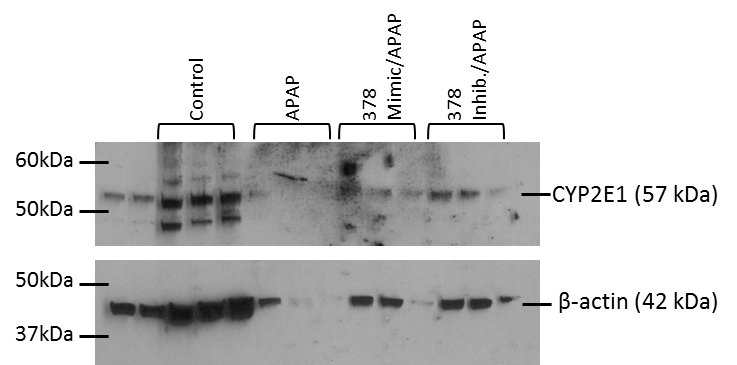
**

**Table S1. miRWalk2.0 predictions of miRNA binding sites for relevant CYPs in APAP toxicity. “√” indicates a binding interaction between CYP gene and miRNA is found.**

| **miRNA** | **Gene** | **Gene regions** | | | | **Specific Algorithms detecting miR-binding sites in 3'UTR** |
| --- | --- | --- | --- | --- | --- | --- |
|  |  | Promoter | 5'UTR | CDS | 3'UTR |  |
| hsa-miR-122-5p | CYP1A1 | √ | √ | √ | √ | RNA22, RNAhybrid |
|  | CYP1A2 | √ |  | √ | √ | RNA22, RNAhybrid |
|  | CYP2E1 | √ |  | √ | √ | RNAhybrid |
|  | CYP3A4 | √ |  | √ | √ | miRWalk2.0, PITA, RNAhybrid, TargetScan |
| hsa-miR-125b-5p | CYP1A1 | √ | √ | √ |  |  |
|  | CYP1A2 | √ |  | √ | √ | RNA22 |
|  | CYP2E1 | √ |  | √ |  |  |
|  | CYP3A4 | √ |  | √ |  |  |
| hsa-miR-27b-3p | CYP1A1 |  | √ | √ | √ | miRMap, PITA, RNAhybrid |
|  | CYP1A2 | √ |  | √ |  |  |
|  | CYP2E1 | √ |  | √ |  |  |
|  | CYP3A4 | √ |  | √ | √ | miRWalk2.0, MicroT4, miRanda, PITA, RNAhybrid, TargetScan |
| hsa-miR-378a-5p | CYP1A1 | √ | √ | √ | √ | RNA22, RNAhybrid |
|  | CYP1A2 | √ |  | √ | √ | RNAhybrid |
|  | CYP2E1 | √ | √ | √ | √ | MicroT4, miRanda, miRMap, RNAhybrid, TargetScan |
|  | CYP3A4 | √ |  | √ | √ | miRWalk2.0, miRanda, RNAhybrid, TargetScan |
| **Interactions (N)** |  | **15** | **5** | **16** | **11** |  |
